# Chronic pain, depression and cardiovascular disease linked through a shared genetic predisposition: Analysis of a family-based cohort and twin study

**DOI:** 10.1371/journal.pone.0170653

**Published:** 2017-02-22

**Authors:** Oliver van Hecke, Lynne J. Hocking, Nicola Torrance, Archie Campbell, Sandosh Padmanabhan, David J. Porteous, Andrew M. McIntosh, Andrea V. Burri, Haruka Tanaka, Frances M. K. Williams, Blair H. Smith

**Affiliations:** 1 Division of Population Health Sciences, School of Medicine, University of Dundee, Dundee, United Kingdom; 2 Institute of Medical Sciences, University of Aberdeen, Aberdeen, United Kingdom; 3 Generation Scotland, Centre for Genomics and Experimental Medicine, Institute of Genetics & Molecular Medicine, University of Edinburgh, Edinburgh, United Kingdom; 4 Institute of Cardiovascular and Medical Sciences, University of Glasgow, Glasgow, United Kingdom; 5 Centre for Genomics and Molecular Medicine, Institute of Genetics & Molecular Medicine, University of Edinburgh, Edinburgh, United Kingdom; 6 Division of Psychiatry, University of Edinburgh, Edinburgh, United Kingdom; 7 Institute of Psychology, University of Zurich, Zurich, Switzerland; 8 Faculty of Health and Environmental Sciences, Auckland University of Technology, Auckland, New Zealand; 9 Dept of Twin Research and Genetic Epidemiology, King’s College London, London, United Kingdom; 10 Mie Prefectural College of Nursing, Tsu, Mie, Japan; University of Illinois at Urbana-Champaign, UNITED STATES

## Abstract

**Background:**

Depression and chronic pain are the two most important causes of disability (Global Burden of Disease Study 2013). They occur together more frequently than expected and both conditions have been shown to be co-morbid with cardiovascular disease. Although shared socio-demographic risk factors (e.g. gender, deprivation) might explain the co-morbidity of these three conditions, we hypothesised that these three long-term, highly prevalent conditions co-occur and may be due to shared familial risk, and/or genetic factors.

**Methods and findings:**

We employed three different study designs in two independent cohorts, namely Generation Scotland and TwinsUK, having standardised, validated questionnaire data on the three traits of interest. First, we estimated the prevalence and co-occurrence of chronic pain, depression and angina among 24,024 participants of a population-based cohort of extended families (Generation Scotland: Scottish Family Health Study), adjusting for age, gender, education, smoking status, and deprivation. Secondly, we compared the odds of co-morbidity in sibling-pairs with the odds in unrelated individuals for the three conditions in the same cohort. Lastly, examination of similar traits in a sample of female twins (TwinsUK, n = 2,902), adjusting for age and BMI, allowed independent replication of the findings and exploration of the influence of additive genetic (A) factors and shared (C) and non-shared (E) environmental factors predisposing to co-occurring chronic widespread pain (CWP) and cardiovascular disease (hypertension, angina, stroke, heart attack, elevated cholesterol, angioplasty or bypass surgery). In the Generation Scotland cohort, individuals with depression were more than twice as likely to have chronic pain as those without depression (adjusted OR 2·64 [95% CI 2·34–2·97]); those with angina were four times more likely to have chronic pain (OR 4·19 [3·64–4·82]); those with depression were twice as likely to have angina (OR 2·20 [1·90–2·54]). Similar odds were obtained when the outcomes and predictors were reversed and similar effects seen among sibling pairs; depression in one sibling predicted chronic pain in the other (OR 1·34 [1·05–1·71]), angina predicted chronic pain in the other (OR 2·19 [1·63–2·95]), and depression, angina (OR 1·98 [1·49–2·65]). Individuals with chronic pain and angina showed almost four-fold greater odds of depression compared with those manifesting neither trait (OR 3·78 [2·99–4·78]); angina showed seven-fold increased odds in the presence of chronic pain and depression (OR 7·76 [6·05–9·95]) and chronic pain nine-fold in the presence of depression and angina (OR 9·43 [6·85–12·98]). In TwinsUK, the relationship between CWP and depression has been published (R = 0.34, p<0.01). Considering the CWP-cardiovascular relationship, the most suitable model to describe the observed data was a combination of A, C and E, with a small but significant genetic predisposition, shared between the two traits (2·2% [95% CI 0·06–0·23]).

**Conclusion:**

We found an increased co-occurrence of chronic pain, depression and cardiovascular disease in two independent cohorts (general population-based cohort, twins cohort) suggesting a shared genetic contribution. Adjustment for known environmental influences, particularly those relating to socio-economic status (Generation Scotland: age, gender, deprivation, smoking, education; Twins UK: age,BMI) did not explain the relationship observed between chronic pain, depression and cardiovascular disease. Our findings from two independent cohorts challenge the concept of traditional disease boundaries and warrant further investigation of shared biological mechanisms.

## Introduction

The prevalence of painful conditions co-occurring with other chronic diseases has been under-recognised until recently [1, 2]. For example, chronic pain is common, affecting approximately 1 in 5 adults in the general population across Europe [3]. Chronic pain with sleep disturbance [4] shows a strong association with anxiety and depression [5] and longitudinal studies suggest the associated overall disease burden among individuals with co-morbidities, co-prescribing and co-occurrence of disability all lead to greater challenges in managing each condition in turn [6].

Observational evidence suggests some form of co-occurrence between chronic pain, depression and/or cardiovascular disease and associated poor health and premature death [2]. This is supported by good bi-directional evidence linking chronic pain and depression [7, 8], depression and cardiovascular disease [9–11], and to a lesser extent, chronic pain and cardiovascular disease [12]. However we are less sure the extent to which one condition is driven by one (or multiple) condition(s), whether co-morbid conditions share a common aetiology, or whether they occur independently.

This co-occurrence of conditions may be related to alterations in the stress-response system [13], and/or metabolic or inflammatory processes [14], or for other reasons yet to be defined, such as genetic predisposition [15]. Because of the complexity of the pain experience and its multi-factorial effect on health-related quality of life, it remains unclear the extent to which the clustering of chronic pain, depression and cardiovascular disease in individuals is the result of a shared aetiology, and/or modifying or confounding factors such as health inequality, as all three conditions are associated with indicators of (relative) deprivation [16].

In this study, we firstly aimed to quantify the likelihood of these conditions co-occurring in a large general population-based cohort, secondly to ascertain whether this may be due in part to shared familial risk, and finally to confirm findings in a UK twin cohort.

## Methods

### Overall study population and participants

#### Generation Scotland

Data were obtained from Generation Scotland: Scottish Family Health Study (GS:SFHS; www.generationscotland.org) [17], a large general population-based cohort (n = 24,042) of extended families recruited across Scotland during 2006–2011.

Data on chronic pain, cardiovascular and mental health variables including depression were collected at the initial study visit by validated self-completed questionnaires and a clinic-based examination. Socio-demographic data included age at assessment, gender, educational attainment and smoking status, and the Scottish Index of Multiple Deprivation 2009 (SIMD), based on residential postcode. SIMD is grouped into quintiles ranking those areas from most deprived (ranked 1) to least deprived (ranked 5) [18]. Participants were unaware of the current study aims. A detailed description of GS:SFHS, including recruitment, data collection and baseline epidemiology, has been published previously [19].

#### TwinsUK

The replication sample was drawn from the TwinsUK registry (www.twinsuk.ac.uk), which has been shown to be representative and comparable to the general population in terms of behavior, lifestyle factors and diseases [20]. Detailed information on the twin cohort has been published elsewhere [21, 22]. Because of the very small number of males with data on CWP and to avoid gender as an additional confounding variable, only females were included in the sample. Zygosity was confirmed by genotyping [23]. A total of 2,902 female twins aged ≥ 17 years, comprising 749 monozygotic (MZ) twin pairs and 702 same-sex dizygotic (DZ) twin pairs were included in this study. Collection of chronic pain phenotypes and genetic information (from blood samples) was carried out during clinical visits and via postal questionnaires. The twin subjects were unaware of the research interests of the present study.

### Variables

#### Chronic pain

In the GS:SFHS cohort, chronic pain was defined as reported pain or discomfort persisting longer than 3 months [24], identified by a validated questionnaire [25], and included the body site(s) affected and site of worst pain [26, 27].The Chronic Pain Grade (CPG) questionnaire was used to assess pain severity based on intensity and pain-related disability in the previous three months [28]. The CPG is a 7-item instrument that classifies severity into four hierarchical grades: Grade I (low disability-low intensity), Grade II (low disability-high intensity), Grade III (high disability-moderately limiting) and Grade IV (high disability-severely limiting. We defined clinically significant chronic pain as those with CPG II-IV, representing those with high pain intensity and/or high pain-related disability. “Chronic pain controls” were those who reported no current pain or discomfort. Participants with non-chronic pain or CPG I were excluded from analyses.

In TwinsUK, the London Fibromyalgia Epidemiology Symptom Screening Questionnaire (LFESSQ) was used to screen for self-reported chronic widespread pain (CWP)[29]. This questionnaire includes four items relating to widespread pain (and two items relating to fatigue). We assessed CWP according to the four items pertaining to the “pain subscale; asking about pain on the left and right sides of body and above and below the diaphragm, and lasting at least seven days in the previous three months. In order to be classified as having CWP, participants had to respond “yes” to all four pain items demonstrating pain on both sides of the body and above and below the diaphragm. This phenotype definition is supported by the contribution this sample has made to previous large studies [30, 31].

#### Depression

All participants in GS:SFHS who attended the research clinic (n = 21,473) were screened for a history of emotional and psychiatric disorders using two questions from the Structured Clinical Interview for DSM-IV Disorders (SCID)[32], a validated instrument for detecting a lifetime history of mental illness. Those who screened positively were invited to complete the full SCID interview conducted face-to-face by a trained health professional. Cases of depression were defined as those identified as ever having had an episode of major depression (single or recurrent). “Depression controls” were those who responded negatively to both SCID screening questions.

In the TwinsUK cohort, the diagnosis of depression was obtained from the Composite International Diagnostic Interview (CIDI) questionnaire according to the DSM-IV criteria for major depression disorder (MDD) and was handled as a dichotomous variable in all analyses [33].

#### Cardiovascular parameters

In the GS:SFHS cohort, angina was identified using the shortened World Health Organisation (WHO) Rose Angina Questionnaire [34], which is used widely in epidemiological studies as a standardised method for identifying angina and as a risk assessment tool for ischaemic heart disease [35]. Population-based studies have shown that individuals with Rose questionnaire angina have an increased risk of incident ischaemic heart disease and more coronary atherosclerosis [36]. The full version of the questionnaire was included in the original GS:SFHS questionnaire. We used the shortened Rose Angina Questionnaire to maximise the availability of complete data (Box 1). This shortened version contains three questions to predict ‘*exertional chest pain’* and has been shown to perform better than the full version at identifying angina in primary care [34].

**Box 1. Shortened WHO Rose Angina questionnaire focusing on exertional chest pain (from Lawlor et al.[34]**

Q1. Do you ever have any pain or discomfort in your chest? (Yes/No)
Q2. When you walk at an ordinary pace on the level does this produce the pain? (Yes/No/Unable)
Q3. When you walk uphill or hurry does this produce the pain? (Yes/No/Unable)

We also included participants who self-reported having ischaemic heart disease, to detect those with well-controlled (medicated) angina who might respond negatively to the Rose Angina questionnaire. Angina was therefore defined by answering ‘Yes’ to question 1 and ‘Yes’ to either question 2 or 3 (Box 1) on the shortened Rose Angina Questionnaire, and/or by answering positively to self-reported ischaemic heart disease. Those who responded negatively to both the shortened Rose Angina questionnaire, and ischaemic heart disease were classified as “angina controls”.

In TwinsUK, a generic variable as an indicator of cardiovascular health/status was used. Participants reporting a medical diagnosis of hypertension, angina, stroke, heart attack, elevated cholesterol, or having had an angioplasty or bypass surgery were assigned as having “any cardiovascular disease (CVD)” (assigned = 1), whereas participants without any such diagnosis were assigned = 0 (no CVD).

### Data analysis

#### Generation Scotland

Data were analysed using SPSS (version 22; SPSS Inc., Chicago, IL, USA) and included only those individuals with valid case/control definitions for each of (i) chronic pain, (ii) major depressive disorder, and (iii) angina, as appropriate for the analysis.

#### Co-occurrence of depression, angina and chronic pain in GS:SFHS (entire cohort)

Individuals were classified according to pairwise co-occurrence of two conditions (chronic pain-depression; chronic pain-angina; depression-angina), or co-occurrence of all three conditions. Co-occurrence controls had neither/none of the conditions under examination. We compared the expected co-occurrence of conditions by chance with the observed co-occurrence prevalence. Stepwise logistic regression was carried out to estimate odds ratios (ORs) with and without adjustment for covariates (age, gender, smoking status, education, deprivation). Covariates with P>0·1 were removed from analysis. Analysis was also stratified by gender. We tested for homogeneity of ORs between genders (Breslow-Day).

Analyses were conducted for the entire cohort (including related individuals), and for a subset of unrelated individuals. Unrelated individuals within pedigrees were identified by selecting the oldest phenotyped family member and removing all biological relatives (e.g. parent, child, sibling, avuncular), then selecting the oldest remaining unrelated, phenotyped pedigree member and repeating this process until only unrelated individuals from within each pedigree remained.

We conducted sensitivity analysis of participants with chronic pain and angina in order to assess the confounding effect of angina also being reported as chronic pain, by identifying those individuals reporting the chest as the main site of chronic pain with angina cases and repeating the analysis excluding these participants.

#### Chronic pain, depression and angina amongst GS:SFHS sibling-pairs

Within each family, full siblings were identified based on reported shared parentage. Where there were more than two siblings with phenotype data available, two siblings were selected at random (independently of all variables included in analyses), and randomly allocated to be “sib1” or “sib2”.

Logistic regression was performed as above, but using as predictor sib1 disease status and outcome sib2 disease status (with and without adjustment for sib2 covariates), and vice versa, for all sibling pairs or within same-gender pairs. Sibling recurrence risk ratios [λ_S_] were calculated from trait prevalence data for the sibling pairs compared with all siblings in the data set (the eligible general population). Analyses were performed for co-occurrence of the same trait in siblings or for co-occurrence of different traits in siblings (cross-phenotype analysis). Cross-phenotype logistic regression was performed with additional adjustment for the same trait in sib2 as the sib1 predictor trait (e.g. when assessing risk for sib2 of having depression when sib1 has chronic pain, analyses were also adjusted for presence of chronic pain in sib2). Results were considered significant at p<0·05.

#### Twins UK

Analyses were conducted by R statistical software version 3.1.2 [37] with bivariate genetic analyses conducted using “OpenMx” packages [38]. First, tetrachoric correlations between CVD, depression, and CWP were calculated. The phenotypic covariance between CVD and CWP was decomposed into the additive genetic factor (A), non-additive genetic factor (D) or shared environmental factor (C), and non-shared environmental factor (E), based on the usual assumptions (i.e. MZ twin pair share all their genes and DZ twin pair share half their genes) [30, 39]. The phenotypic covariance between the observed variables was partly decomposed into A and D when the phenotypic covariance in MZ twin pairs was higher than in those in DZ [30]. When the phenotypic covariance in MZ twin pairs was similar to those in DZ, the phenotypic covariance was decomposed into C.

**Fig 1:**
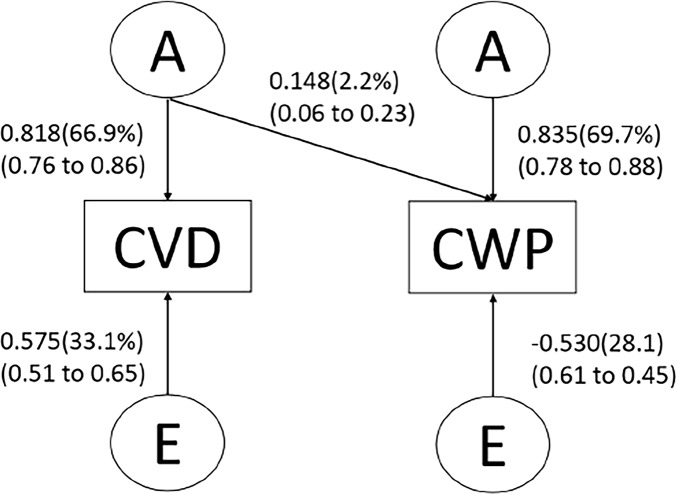
Path diagram of the Cholesky model for the covariance between chronic widespread pain (CWP) and cardiovascular disease (CVD) with adjustment for age.

To identify the best fitting model, a three-step approach was conducted using structural equation modelling. First, we compared the full ACE and ADE bivariate Cholesky model with fully saturated model because C and D could not be estimated in the same model. Fig 1 shows the full ACE Cholesky model. The variance‐covariance matrix in the fully saturated model was treated as a free parameter that was equal to the sample variance‐covariance matrix. Second, we compared the full ACE or ADE bivariate Cholesky model with the AE bivariate Cholesky model to test whether liquidation of C or D factors was legitimate. Finally, we explored the best fitting submodels by eliminating parameters and latent variables and by comparing these submodels with the full ACE or ADE bivariate Cholesky model. The best fitting model was determined on the basis of the likelihood ratio test and the lowest AIC (Akaike information criterion). All phenotypes were controlled for age and body mass index. Standardized coefficients and 95% confidence intervals (CIs) were obtained. To handle missing data, full-information maximum likelihood was used. Under the missing at random assumption, full-information maximum likelihood was able to provide preferred parameter estimates [40].

### Ethics and approval

GS:SFHS received ethical approval for the creation of the resource (05/S1401/89 NHS Tayside Committee on Medical Research Ethics A) and Research Tissue Bank approval has been granted providing for use of the data and samples for medical research, including this study (10/S1402/20 and 15/ES/0040 NHS EoSRES). All participants provided written informed consent.

TwinsUK had received ethical approval from the Westminster ethics committee (07/H0802/84) and all participants provided written informed consent.

### Funding

Generation Scotland has received core funding from the Chief Scientist Office of the Scottish Government Health Directorates CZD/16/6 and the Scottish Funding Council HR03006. The funders had no role in study design, data collection, data analysis, data interpretation, or writing of the report, or the decision to submit for publication. The corresponding author had final responsibility for the decision to submit for publication.

TwinsUK: the study was funded by the Wellcome Trust; European Community’s Seventh Framework Programme (FP7/2007-2013). The study also receives support from the National Institute for Health Research (NIHR)-funded BioResource, Clinical Research Facility and Biomedical Research Centre based at Guy's and St Thomas' NHS Foundation Trust in partnership with King's College London.

## Results

### Characteristics of study population: Generation Scotland

The characteristics of the entire study population, unrelated individuals and sibling pairs are shown in Table 1. Of the 24,042 participants in the GS:SFHS cohort, the majority were female (58·7%). The median age was 49 years (IQR 36–59) and just over one-quarter of the cohort lived in the two most deprived SIMD quintiles. Compared to the “unrelated” subgroup, the overall population cohort and sibling-pairs subgroup were most alike across most demographic characteristics. Those in the “unrelated” group were older, likely due to the method used to select unrelated individuals.

**Table 1:** Characteristics of the overall GS:SFHS, unrelated subset and sibling pairs subset^‡^.

	Overall GS:SFHS	Unrelated individuals	Sibling pairs
	n = 24,042	n = 9,163	n = 11, 740
**Age (median, IQR)**	49 yr (IQR 36–59)	59 yr (52–65)	47 yr (IQR 36–57)
**Gender (female)**	14,064 (58·7%)	5,353 (58·4%)	7,011 (59·9%)
**Education**			
Degree, diploma or technical qualification	13,741 (61·7%)	4,663 (55·7%)	7,292 (65·1%)
School leaving	6,043 (27·1%)	2,287 (27·3%)	2,873 (25·6%)
No qualification	2,497 (11·2%)	1,420 (17%)	1,039 (9·3%)
**Deprivation (SIMD)**			
1 (most deprived)	2,711 (12·9%)	1,052 (12·7%)	1,297 (12·7%)
2	2,965 (14·1%)	1,126 (13·6%)	1,363 (13·3%)
3	3,417 (16·3%)	1,307 (15·8%)	1,666 (16·3%)
4	5,392 (25·7%)	2,059 (24·8%)	2,698 (26·4%)
5 (least deprived)	6,529 (31·1%)	2,749 (33·1%)	3,186 (31·2%)
**Smoking status**			
Never smoked	12,267 (52·7%)	4,527 (51·0%)	6,339 (54·6%)
Stopped >12 months	6,279 (27·0%)	2,925 (32·9%)	2,955 (25·5%)
Stopped < 12 months	713 (3·1%)	182 (2·0%)	351 (3·0%)
Current smoker	4,013 (17·2%)	1,249 (14·1%)	1,963 (16·9%)
**Any chronic pain (CPG I–IV)**	7,162 (35·5%)	3,106 (42·3%)	3,665 (34·0%)
**Clinically-significant chronic pain (CPG II–IV)**	3,664 (18·1%)	1,641 (27·9%)	1,755 (19·8%)
**Major depressive disorder (MDD)**	2,755 (12·9%)	960 (12·8%)	1,513 (15·0%)
**Angina**	2,009 (10·0%)	1,102 (14·4%)	851 (8·0%)

^‡ ^Percentages (%) is based on valid data; SIMD: Scottish Index of Multiple Deprivation 2009; CPG: chronic pain grade

#### Chronic pain

In total, 20,199 individuals completed the chronic pain screening questions. There were 7,162 individuals for whom a CPG was calculated (35·5%) and 13,037 “chronic pain controls”. Of those for whom the CPG was calculated, 3,498 (17·3%) were classed as CPG I; 2,438 (12·1%) as CPG II; 611 (3·0%) as CPG III, and 615 (3·0%) as CPG IV, making 3,664 (18·1%) individuals with clinically significant chronic pain (CPG II-IV) who were classed as “chronic pain cases”.

#### Depression

Of those who attended the research clinic, 21,380 had valid depression screening results. Just over one-fifth (n = 4,714) screened positive and were invited to complete the SCID interview, leaving 16,666 individuals as “depression controls”. Of the screened individuals, 84·2% (n = 3,968) completed the face-to-face clinical interview, where 2,755 individuals (12·9%) were identified as “depression cases”. Those with bipolar mood disorder or no history of major depressive disorder following SCID interview were excluded from further analysis (n = 1,213).

#### Angina

Of the 20,115 valid responses to the short Rose angina questionnaire 1,684 (8·4%) individuals were identified as cases of angina. There were 930 (4·0%) who volunteered a history of ischaemic heart disease, 325 of whom had screened negative to the Rose Angina questionnaire. Thus there were 2,009 “angina cases” (10·0%) and 18,106 “angina controls”.

### Co-occurrence of depression, angina and chronic pain: Generation Scotland

The co-occurrence of chronic pain and depression (Table 2) was seen in 5·3%, (714/13,422 of the GS:SFHS cohort with valid responses); chronic pain and angina 4·6%, (678/14,616); and depression and angina 2·3% (371/16,284). All three conditions co-occurred in 1·8% of those with valid responses (169/9,492). All conditions co-occurred more often than expected by chance: expected frequency of chronic pain and depression co-occurrence 2·3% vs 5·3% observed co-occurrence; chronic pain and depression 1·8% vs 4·6%; depression and angina 1·2% vs 2·3%.

**Table 2:** Co-occurrence of chronic pain, major depressive disorder and/or angina in GS:SFHS overall.

Combinations of conditions	Presence of conditions ^1^	Absence of conditions ^2^	Total
Chronic pain and depression	714 (5·3%)	9,424	13,422
Chronic pain and angina	678 (4·6%)	11,058	14,616
Depression and angina	371 (2·3%)	12,880	16,284
Depression, angina and chronic pain	169 (1·8%)	8,089	9,492

^1 ^**Presence of conditions**: Chronic pain = Chronic Pain Grade II-IV; Depression = positive after SCID interview as ever having had an episode of major depressive disorder (single or recurrent); Angina = positive for shortened Rose angina questionnaire or self-reported history of ischaemic heart disease

^**2**^ **Absence of conditions:** Chronic pain = no current pain to screening questions; Depression = negative to both SCID screening questions; Angina = negative to shortened Rose Angina questionnaire, and no history of ischaemic heart disease.

Subgroup analysis of chronic pain and angina (n = 678) showed that individuals with angina reported numerous sites of chronic pain. Individuals with angina and chronic pain reported their most painful body site as follows: pains in arms, hands, legs, hips or feet (n = 170); back pain (n = 124); neck and shoulder pain (n = 70); chest pain (n = 47). Of the latter 47 participants, only 14 reported ‘chronic chest pain’ as their sole location of chronic pain. Sensitivity analysis excluding those with angina and the chest as their main site of chronic pain found no alteration in the final results.

### Association between chronic pain, depression and angina: Generation Scotland

Individuals with depression were more than twice as likely to have chronic pain as those without after adjustment for known confounders (adjusted OR 2·64 [95% CI 2·34–2·97]), while those with angina were four times more likely to have chronic pain (adjusted OR 4·19 [3·64–4·82]) (Table 3). Individuals with both angina and depression had greater odds of also having chronic pain (adjusted OR 9·43 [6·85–12·98]. Female participants had slightly greater ORs than male participants, but none of the differences were significant (Breslow-Day P>0·05) (S1 Table).

**Table 3:** The effect of comorbidity on the occurrence of depression and/or angina in GS:SFHS overall.

Exposure	Outcome	Unadjusted		Adjusted^†^	
		N	OR [95% CI]	N	OR [95% CI]
**Chronic pain in the presence of depression**					
Depression	Chronic pain	13, 376	2·80 ^a^	11,679	2·64^a^
			[2·52–3·11]		[2·34–2·97]
**Chronic pain in the presence of angina**					
Angina	Chronic pain	14,564	5·44 ^a^	11,973	4·19^a^
			[4·83–6·13]		[3·64–4·82]
**Chronic pain in the presence of depression and angina**					
Depression and angina	Chronic pain	9,728	13·28 ^a^	8,543	9·43^a^
			[10·02–17·59]		[6·85–12·98]
**Depression in the presence of chronic pain**					
Chronic Pain	Depression	13,376	2·80^a^	11,679	2·62^a^
			[2·52–3·11]		[2·32–2·96]
**Depression in the presence of angina**					
Angina	Depression	16,224	2·18^a^	14,121	2·10^a^
			[1·92–2·47]		[1·82–2·43]
**Depression in the presence of chronic pain and angina**					
Chronic pain and angina	Depression	9,461	4·36^a^	8,297	3·78 ^a^
			[3·58–5·30]		[2·99–4·78]
**Angina in the presence of chronic pain**					
Chronic pain	Angina	14,564	5·45^a^	11,973	4·23 ^a^
			[4·84–6·14]		[3·67–4·86]
**Angina in the presence of depression**					
Depression	Angina	16,224	2·18^a^	14,121	2·20 ^a^
			[1·92–2·47]		[1·90–2·54]
**Angina in the presence of chronic pain and depression**					
Chronic pain and depression	Angina	9,003	9·15^a^	7,919	7·76 ^a^
			[7·43–11·26]		[6·05–9·95]

^†^valid data adjusted for age, gender, education, SIMD and smoking status

^a^ = p<0.001.

Reversing the analyses (Table 3), we found that individuals with chronic pain were more likely to have a history of depression than not (adjusted OR 2·62 [2·32–2·96]) and were more likely to report angina symptoms than not (adjusted OR 4·23 [3·67–4·86]). Similarly, individuals with depression were more likely to have angina than not (adjusted OR 2·20 [1·90–2·54]). Individuals with both depression and chronic pain were much more likely to report angina symptoms than not (adjusted OR 7·76 [6·05–9·95]). In the presence of chronic pain, female participants had higher ORs than male participants for depression and angina respectively, but none of the differences were significant (Breslow-Day P>0·05) (S2 Table). Results from the “unrelated” subgroup (S3–S5 Tables) were similar to the larger family-based cohort, indicating that the associations between chronic pain, depression and angina in the entire cohort were not due to excess shared familial factors (genetic or environmental) in the family-based study.

### Chronic pain, depression and angina amongst sibling-pairs: Generation Scotland

Sib-pair analysis (S7 Table) produced similar ORs to both the entire cohort and the “unrelated” subset for unadjusted and adjusted analyses, and for gender-stratified analysis, suggesting that no bias was introduced during sib selection.

For the same-trait analysis, there was a two-fold increased risk to sib2 for chronic pain when sib1 had chronic pain (adjusted OR 2·30 [1·83–2·89]). This increased risk for the same trait was apparent with depression (adjusted OR 2·16 [1·73–2·70]) and angina (adjusted OR 2·78 [2·00–3·86]) (S6 Table). Results were similar with same-gender sib pairs (S8 Table). Consistent with these findings, sibling recurrence risk ratios (λ_S_) significantly exceeded 1 for all same-trait comparisons, with λ_S_ corresponding to odds ratios. For depression, λ_S_ = 1·65; chronic pain λ_S_ = 1·84; and angina λ_S_ = 2·31 (S6 Table).

In cross-trait analyses, the sib risk was significantly increased in all pairwise comparisons (pain-depression; angina-pain; depression-angina), with adjusted ORs ranging from 1·34–2·19 (Table 4). ORs were unaffected or only slightly attenuated after adjustment; and were largely similar for same-gender sib pairs, although p values attenuated owing to reduced sample size (S9 Table). Additional adjustment for the same trait in sib2 as present for sib1 had little effects on the results, indicating that the increased risk to sib2 was not attributable to presence of the same trait as present for sib1. This finding suggests that the increased risk was instead due to presence of a common risk factor for both disorders. Cross-trait λ_S_ also significantly exceeded 1 for all pairwise comparisons, ranging from 1·24 (depression-chronic pain) to 1·83 (angina-chronic pain), consistent with shared familial risk factors in these disorders.

**Table 4:** Unadjusted and adjusted ORs for cross-trait analysis of angina, depression and chronic pain amongst sibling-pairs in GS:SFHS.

Exposure (Sib1 status)	Outcome (Sib2 status)	Unadjusted		Adjusted		Also adjusted for sib1 trait in sib2		λ_S_
		N	OR [95% CI]	N	OR [95% CI]	N	OR [95% CI]	
Angina	Chronic pain	4,014	2·61	3,275	2·19	3,006	1·66	1·83
			[2·03–3·37] ***		[1·63–2·95] ***		[1·19–2·33] **	[1·56–2·15] *
Angina	Depression	4,595	1·52	3,967	1·48	3,581	1·45	1·35
			[1·16–1·99] **		[1·09–2·01] **		[1·04–2·02] *	[1·09–1·66] *
Chronic pain	Angina	4,026	2·48	3,280	2·01	2,530	1·99	1·71
			[1·93–3·19] ***		[1·49–2·71] ***		[1·38–2·86] ***	[1·42–2·07] *
Chronic pain	Depression	3,831	1·84	3,295	1·69	2,504	1·71	1·43
			[1·50–2·26] ***		[1·34–2·14] ***		[1·28–2·28] ***	[1·24–1·66] *
Depression	Angina	4,562	1·97	3,757	1·98	3,414	1·96	1·70
			[1·54–2·54] ***		[1·49–2·65] ***		[1·44–2·67] ***	[1·39–2·08] *
Depression	Chronic pain	3,796	1·30	3,124	1·34	2,842	1·31	1·24
			[1·05–1·61] *		[1·05–1·71] *		[1·00–1·70] *	[1·06–1·44] *

λ_S_ = sibling recurrence risk ratio

*p ≤0.05

** p ≤0.01 &

*** p ≤0.001

### Characteristics of study population: TwinsUK

Table 5 summarises the main characteristics of the TwinsUK cohort. The mean age was 56 years old (SD 13·97). The proportion of participants with chronic pain was 20%, 22·3% had depression and 34·7% reported any cardiovascular disease. No significant association between depression and cardiovascular disease could be detected, whereas chronic pain showed a significant correlation with depression (r = .34) and cardiovascular disease (r = .26; Table 6).

**Table 5:** Study characteristics of the n = 2,902 twins from the TwinsUK cohort.

	Overall (n = 2,902)	MZ (n = 1,498)	DZ (n = 1,404)
Age, mean (SD), range	56 yr (13.97), 18–89	55 yr (14.92), 18–86	57 yr (12.79), 20–89
BMI, mean (SD), range	25 (4.5), 15–53	25 (4.46),15–53	25 (4.54), 15–48
Cardiovascular disease, n (%)	1,006 (34.7%)	473 (31.6%)	533 (38.0%)
Depression, n (%)	646 (22.3%)	341 (22.8%)	305 (21.7%)
Chronic pain^±^, n (%)	579 (20.0%)	265 (17.7%)	314 (22.4%)

MZ, monozygotic; DZ, dizygotic; SD, standard deviation; BMI, body mass index; ^±^chronic widespread pain

**Table 6:** Phenotypic correlations (r) with [95% confidence intervals] between observed variables in TwinsUK (n = 2,902).

	Depression	CVD	CWP
Depression	1		
Cardiovascular disease	0.04 [-0.01–0.08]	1	
Chronic pain^±^	0.34 [0.30–0.37]	0.26 [0.23–0.29]	1

^±^chronic widespread pain.

### Interclass correlation and cross-twin cross-trait correlation: TwinsUK

The intraclass correlations were consistently higher in MZ compared to DZ twin pairs across all three phenotypes (Table 7). Furthermore, significant cross-trait cross-twin correlation could be detected in the MZ twins but not in the DZ twins.

**Table 7:** Intraclass correlations and cross twin trait correlations [95% confidence interval] in Twins UK.

	MZ	DZ
**Cross-twin/within trait**		
Depression	0.51 [0.44–0.58]	0.20 [0.11–0.3]
Cardiovascular disease	0.75 [0.72–0.78]	0.51 [0.46–0.57]
Chronic pain^**±**^	0.73 [0.69–0.76]	0.46 [0.39–0.51]
**Cross-twin/cross-trait**		
Depression/Cardiovascular disease	0.12 [0.03–0.20]	-0.02 [-0.11–0.07]
Depression/Chronic pain^**±**^	0.22 [0.14–0.30]	0.19 [0.10–0.28]
Cardiovascular disease/Chronic pain^**±**^	0.27 [0.20–0.34]	0.16 [0.09–0.23]

^±^chronic widespread pain; MZ, monozygotic; DZ, dizygotic.

### Bivariate Cholesky model: Twins UK

We were unable to obtain well-fitting models for the trivariate analysis. Thus having published already on the relationship between chronic pain and depression [41], we concentrated on the bivariate analysis of chronic pain and cardiovascular disease. In the saturated models, the mean and variance of all phenotype were equal across twin order and zygosity, so met the requirement for bivariate Cholesky models. On the basis of the AIC, the ACE Cholesky model was considered to be the best fitting model (Table 8). Dropping C resulted in an even better model fit (S10 Table), suggesting that common genetic factors and independent non-shared environmental factors contribute to the covariation in chronic pain and cardiovascular disease (S10 Table and Fig 1).

**Table 8:** Model comparison between the full saturated, the full ACE Cholesky, and the full ADE Cholesky models for chronic pain^±^ and cardiovascular disease (n = 2,902): Twins UK.

	Difference of log likelihood	Difference of degree of freedom	P-value	AIC	BIC
Full saturated model	Base	Base	NA	-5906.552	-36361.64
Full ACE Cholesky model	37.14781	25	0.06	-5919.404	-36506.49
Full ADE Cholesky model	42.37443	25	0.02	-5914.177	-36501.26

A, additive genetic factors; C, shared environmental factors; E, nonshared environmental factors; D, non-additive genetic factors

^±^chronic widespread pain; AIC, Akaike information criterion; BIC, Schwarz’s Bayesian information criteria; NA, not applicable.

## Discussion

This is the first, large-scale study to examine the three important traits of chronic pain, cardiovascular disease and depression, where the findings have been reproduced to some extent in a second, independent population sample. While a genetic component has long been recognised in each of the three conditions individually, it has been thought that social risk factors such as poverty and deprivation were likely to underlie their co-occurrence. To our surprise, even adjusting for socio-economic status and other environmental risk factors, we found significant odds ratios for co-occurrence. This led us to model chronic pain and cardiovascular disease in twins, and to show a small but significant shared genetic predisposition.

We have shown that three common long-term conditions: chronic pain, cardiovascular disease and/or depression cluster together in two epidemiologically unbiased samples. Our family-based study brings new insight into the contribution of shared familial factors to this co-occurrence, showing that this association clearly navigates across conditions within sibling-pairs: the presence of one condition increases the risk of other conditions occurring, both in an individual and in his/her family members. Together with the results from the twins’ analysis, this supports the notion that shared, genetically mediated aetiological pathways contribute to the co-morbidity of common chronic conditions.

A number of common biological pathways have already been implicated in development of depression, chronic pain or cardiovascular disease in experimental models of disease and in human studies. These include the endocannabinoid system, hypothalamic-pituitary-adrenal (HPA) axis, and inflammation. Genetic modification of the endocannabinoid system leads to age-related ventricular dysfunction [42], while genetic variants in cannabinoid and/or adrenergic pathway components have been implicated in chronic pain conditions [43, 44]. Brain expression of endocannabinoid-related genes are altered in response to early life stress [45], and this system may act to sensitise excitatory neurotransmission [46]. HPA axis dysfunction in offspring as a result of maternal stress during pregnancy has also been described [47] as well as early life stress [48, 49]. Inflammatory pathways have also been implicated in development of depression [50], chronic pain [51], and cardiovascular disease [52], in particular the concept that adverse environmental factors (including early life stress) might transmit a biological signal via altered allostatic load, leading to multimorbidity in adulthood (including chronic pain, mental health and cardiovascular conditions) [53]. However, whether these are epiphenomena versus causal remains unclear [51, 54] but new techniques such as Mendelian randomisation are allowing direction of causation to be determined, such as the adrenal hormone abnormalities appearing secondary to CWP [55]. A genetic liability for all three disorders is widely reported, and may also explain the familial component to co-occurrence of these three traits; identification of a shared genetic predisposition and assessment of whether these are shared across multiple disorders is required.

There are limitations to the study to be aware of when interpreting our findings. First, diagnosis of “cardiovascular disease” in both datasets relied on self-reported data and/or questionnaire using well-validated instruments [24, 32]. Specifically in TwinsUK, this was relatively non-specific and included a number of risk factors such as hypercholesterolaemia. This would have served, however, to weaken our findings rather than strengthen them, so it is all the more surprising that a shared genetic influence was detected between cardiovascular disease and chronic pain. The small number of males with chronic pain and CVD data in TwinsUK meant that only females could be considered; however, the large population-based Generation Scotland cohort shows that the relationship between the traits is seen both in men and in women.

We recognise that there are differences in phenotyping, data collection, case definitions and measures used between the two studies, and this is a limitation of aligning two distinct but complimentary studies. In particular, the definitions of cardiovascular disease differed between GS:SFHS and TwinsUK, and comparison between the cohorts can only be approximate in this phenotype. However, the phenotyping is similar enough to allow comparison between these two population-based studies. The handling of covariates is a limitation, with BMI not included in GS, and with modelling in TwinsUK not allowing for inclusion of covariates such as smoking or education; alignment of the two cohorts is therefore incomplete.

We had no means of identifying people who had a history of chronic pain but who were not currently affected, and these would have been included as controls. However, clinical experience suggests that the proportion of patients who have chronic pain and get better is very small indeed. We did not distinguish past major depression from that which was current. Nonetheless it seems that depression in “remission”, i.e. a *history* of major depressive disorder, still confers a significant risk of developing the linked condition e.g. chronic pain [56, 57]. As with all cross-sectional studies, the directional relationships between these three conditions cannot be determined, pointing to the need for longitudinal studies. Similarly, the relationship with other chronic diseases co-morbid with these conditions (e.g. type 2 diabetes) was not explored.

Information on specific medications taken by individuals to manage each condition in our datasets were not available. Potentially this could lead to under-reporting of a condition (e.g. chronic pain) which is well controlled by medications. Any future study should aim to correlate self-reported angina, chronic pain and depression with electronic health records and relevant prescribed medication. While medication can *reduce* the severity and impact of chronic pain or angina, it is unlikely to *remove* it–the diagnosis is therefore still likely to be self-reported, though its reported severity may be less than were it untreated.

The possibility that some other shared environment not included in our model and not captured by current deprivation status has confounded the results, is made considerably less likely by the results from the twin study. We have already reported a shared genetic influence on the risk of chronic pain and depression in TwinsUK [41]. The twin sample size limited our ability to perform three-way analysis.

Clinically, depression and cardiovascular disease are well-known co-morbidities [58], prompting routine screening in primary care cardiovascular clinics [59]. The demonstration that the presence of chronic pain may increase the likelihood of depression and/or cardiovascular disease has further implications for clinical practice, not least because it is more prevalent than both depression (9%) or ischaemic heart disease (8·3%)[60]. The reported chronic pain prevalence across Europe is around 20% [3], and another study in the UK suggests this is around 24% (CPG II-IV) [61]. That almost a quarter of the UK population manifest this important risk factor for cardiovascular disease and depression should prompt further screening in patients and their family members.

These results have far-reaching implications for targeted management and prevention, and for prognosis. Might effective treatment of one condition ameliorate a co-occurring, linked condition? Future clinical trials might, very cost-effectively, explore whether optimising depression therapy in chronic pain leads to improved outcomes [62], and this could extend to family members. Early recognition of these conditions will facilitate trials of prevention and also the potential for addressing these conditions by targeting the common underlying causes (e.g. stress, health inequalities) rather than symptoms unique to each condition.

## Conclusion

We have shown that chronic pain, cardiovascular disease, and depression coexist in individuals and across families even after adjusting for known environmental risk factors. The presence of one trait significantly increases the risk of manifesting another, both in siblings and twins. Pairs of traits offer even higher risks of manifesting the third. Twin modelling shows that pairs of traits have shared genetic predisposition which would account for their co-occurrence. Future work should aim to identify the genetic variants involved, as better understanding of the biological pathways will clarify the underlying pathogenetic mechanisms as well as having the potential to provide novel targets for intervention.

## Supporting information

S1 Table The effect of depression and/or angina on the occurrence of chronic pain in GS: SFHS overall and stratified according to gender. (PDF)

S2 Table The effect of comorbidity on the occurrence of depression and/or angina in GS: SFHS overall and stratified according to gender. (PDF)

S3 Table The effect of chronic pain on the occurrence of depression and/or angina in the “unrelated” GS:SFHS subgroup (n = 9,163) and stratified according to gender. (PDF)

S4 Table The effect of depression on the occurrence of chronic pain and/or angina in the GS:SFHS “unrelated” subgroup (n = 9,163) and stratified according to gender. (PDF)

S5 Table The effect of angina on the occurrence of depression and/or chronic pain in the GS:SFHS “unrelated” subgroup (n = 9,163) and stratified according to gender. (PDF)

S6 Table Unadjusted and adjusted ORs for co-occurrence of the same trait within sibling-pairs in GS:SFHS (PDF)

S7 Table Unadjusted and adjusted ORs of angina, depression and chronic pain within sib1 subset, GS:SFHS. (PDF)

S8 Table Unadjusted and adjusted ORs for co-occurrence of the same trait overall and within same-gender sibling-pairs, GS:SFHS. (PDF)

S9 Table Unadjusted and adjusted ORs for cross-trait analysis of angina, depression and chronic pain overall and within same-gender sibling-pairs, GS:SFHS. (PDF)

S10 Table Model comparison between the full ACE Cholesky and the best balance of model fit with parsimony for chronic pain^±^ and cardiovascular disease (n = 2,902): Twins UK. (PDF)

## References

[1] Barnett K, Mercer SW, Norbury M, Watt G, Wyke S, Guthrie B. Epidemiology of multimorbidity and implications for health care, research, and medical education: a cross-sectional study. Lancet. 2012;380(9836):37–43. 10.1016/S0140-6736(12)60240-2 22579043

[2] Dominick C, Blyth F, Nicholas M. Unpacking the burden: Understanding the relationships between chronic pain and co-morbidity in the general population. Pain. 2012;153:292–304. 10.1016/j.pain.2011.09.018 22071318

[3] Breivik H, Collett B, Ventafridda V, Cohen R, Gallacher D. Survey of chronic pain in Europe: prevalence, impact on daily life, and treatment. Eur J Pain. 2006;10(4):287–333. 10.1016/j.ejpain.2005.06.009 16095934

[4] Staud R. Chronic widespread pain and fibromyalgia: two sides of the same coin? Current rheumatology reports. 2009;11(6):433–6. 19922733 10.1007/s11926-009-0063-8

[5] Wolfe F, Michaud K, Li T, Katz RS. Chronic conditions and health problems in rheumatic diseases: comparisons with rheumatoid arthritis, noninflammatory rheumatic disorders, systemic lupus erythematosus, and fibromyalgia. The Journal of rheumatology. 2010;37(2):305–15. 10.3899/jrheum.090781 20080915

[6] van Hecke O, Torrance N, Smith BH. Chronic pain epidemiology–where do lifestyle factors fit in? British Journal of Pain. 2013;7(4):209–17. 10.1177/2049463713493264 26516524 PMC4590163

[7] Von Korff M, Alonso J, Ormel J, Angermeyer M, Bruffaerts R, Fleiz C, et al Childhood psychosocial stressors and adult onset arthritis: broad spectrum risk factors and allostatic load. Pain. 2009;143:76–83. 10.1016/j.pain.2009.01.034 19251363 PMC2703588

[8] Von Korff M, Le Resche L, Dworkin SF. First onset of common pain symptoms: a prospective study of depression as a risk factor. Pain. 1993;55:251–8. 8309712 10.1016/0304-3959(93)90154-H

[9] Charlson FJ, Moran AE, Freedman G, Norman RE, Stapelberg NJ, Baxter AJ, et al The contribution of major depression to the global burden of ischemic heart disease: a comparative risk assessment. BMC medicine. 2013;11:250 10.1186/1741-7015-11-250 24274053 PMC4222499

[10] Goldston K, Baillie AJ. Depression and coronary heart disease: a review of the epidemiological evidence, explanatory mechanisms and management approaches. Clinical psychology review. 2008;28(2):288–306. 10.1016/j.cpr.2007.05.005 17601644

[11] Van der Kooy K, van Hout H, Marwijk H, Marten H, Stehouwer C, Beekman A. Depression and the risk for cardiovascular diseases: systematic review and meta analysis. International Journal of Geriatric Psychiatry. 2007;22(7):613–26. 10.1002/gps.1723 17236251

[12] Torrance N, Elliott A, Lee A, Smith B. Severe chronic pain is associated with increased 10 year mortality. A cohort record linkage study. Eur J Pain. 2010;14(4):380–6. 10.1016/j.ejpain.2009.07.006 19726210

[13] Holliday K, Nicholl BI, Macfarlane GJ, Thomson W, Davies KA, McBeth J. Genetic variation in the hypothalamic-pituitary-adrenal stress axis influences susceptibility to musculoskeletal pain: results from the EPIFUND study. Ann Rheum Dis. 2010;69:556–60. 10.1136/ard.2009.116137 19723618 PMC2927682

[14] Goodson NJ, Smith BH, Hocking LJ, McGilchrist MM, Dominiczak AF, Morris A, et al Cardiovascular risk factors associated with the metabolic syndrome are more prevalent in people reporting chronic pain: Results from a cross sectional general population study. Pain 2013:S0304-3959(13)00221-2. 10.1016/j.pain.2013.04.043 23707277

[15] Stone AL, Wilson AC. Transmission of risk from parents with chronic pain to offspring: an integrative conceptual model. Pain. 2016;157(12):2628–39. 10.1097/j.pain.0000000000000637 27380502 PMC5592972

[16] McLean G, Gunn J, Wyke S, Guthrie B, Watt GC, Blane DN, et al The influence of socioeconomic deprivation on multimorbidity at different ages: a cross-sectional study. The British journal of general practice: the journal of the Royal College of General Practitioners. 2014;64(624):e440–7. 24982497 10.3399/bjgp14X680545 PMC4073730

[17] Smith BH, Campbell H, Blackwood D, Connell J, Connor M, Deary IJ, et al Generation Scotland: the Scottish Family Health Study; a new resource for researching genes and heritability. BMC Med Genet. 2006;7:74 10.1186/1471-2350-7-74 17014726 PMC1592477

[18] Scottish Index of Multiple Deprivation [Available from: http://www.scotland.gov.uk/topics/statistics/simd/

[19] Smith BH, Campbell A, Linksted P, Fitzpatrick B, Jackson C, Kerr SM, et al Cohort Profile: Generation Scotland: Scottish Family Health Study (GS:SFHS). The study, its participants and their potential for genetic research on health and illness. International journal of epidemiology. 2013;42(3):689–700. 10.1093/ije/dys084 22786799

[20] Andrew T, Hart DJ, Snieder H, de Lange M, Spector TD, MacGregor AJ. Are twins and singletons comparable? A study of disease-related and lifestyle characteristics in adult women. Twin research: the official journal of the International Society for Twin Studies. 2001;4(6):464–77. 11780939 10.1375/1369052012803

[21] Moayyeri A, Hammond CJ, Hart DJ, Spector TD. The UK Adult Twin Registry (TwinsUK Resource). Twin research and human genetics: the official journal of the International Society for Twin Studies. 2013;16(1):144–9. 23088889 10.1017/thg.2012.89 PMC3927054

[22] Spector TD, Williams FM. The UK Adult Twin Registry (TwinsUK). Twin research and human genetics: the official journal of the International Society for Twin Studies. 2006;9(6):899–906. 17254428 10.1375/183242706779462462

[23] von Wurmb-Schwark N, Schwark T, Christiansen L, Lorenz D, Oehmichen M. The use of different multiplex PCRs for twin zygosity determination and its application in forensic trace analysis. Legal medicine (Tokyo, Japan). 2004;6(2):125–30. 10.1016/j.legalmed.2003.12.002 15039056

[24] Classification of chronic pain. Descriptions of chronic pain syndromes and definitions of pain terms. Prepared by the International Association for the Study of Pain, Subcommittee on Taxonomy. Pain Supplement. 1986;3:S1–226. 3461421

[25] Purves AM PK, Munro C, Smith BH, Grimshaw J, Wilson B, Smith WC, Chambers WA. Defining chronic pain for epidemiological research—assessing a subjective definition. The Pain Clinic. 1998;10:139–47.

[26] Hocking L, Generation Scotland, Morris A, Dominiczak A, Porteous D, Smith B. Heritability of chronic pain in 2195 extended families. Eur J Pain. 2012;16:1053–63. 10.1002/j.1532-2149.2011.00095.x 22337623

[27] van Hecke O, Torrance N, Cochrane L, Cavanagh J, Donnan PT, Padmanabhan S, et al Does a history of depression actually mediate smoking-related pain? Findings from a cross-sectional general population-based study. Eur J Pain. 2014;18(9):1223–30. 10.1002/j.1532-2149.2014.00470.x 24577799

[28] Von Korff M, Ormel J, Keefe FJ, Dworkin SF. Grading the severity of chronic pain. Pain. 1992;50:133–49. 1408309 10.1016/0304-3959(92)90154-4

[29] White KP, Speechley M, Harth M, Ostbye T. The London Fibromyalgia Epidemiology Study: direct health care costs of fibromyalgia syndrome in London, Canada. The Journal of rheumatology. 1999;26(4):885–9. 10229411

[30] Burri A, Ogata S, Vehof J, Williams F. Chronic widespread pain: clinical comorbidities and psychological correlates. Pain. 2015;156(8):1458–64. 10.1097/j.pain.0000000000000182 25851458

[31] Peters MJ, Broer L, Willemen HL, Eiriksdottir G, Hocking LJ, Holliday KL, et al Genome-wide association study meta-analysis of chronic widespread pain: evidence for involvement of the 5p15.2 region. Ann Rheum Dis. 2013;72(3):427–36. 10.1136/annrheumdis-2012-201742 22956598 PMC3691951

[32] First MB, Spitzer RL, Gibbon M, Williams JBW. Structured Clinical Interview for DSM-IV-TR Axis I Disorders, Research Version, Non-patient Edition. (SCID-I/NP) New York: New York State Psychiatric Institute, November 2002.

[33] Kessler RC, Ustun TB. The World Mental Health (WMH) Survey Initiative Version of the World Health Organization (WHO) Composite International Diagnostic Interview (CIDI). International journal of methods in psychiatric research. 2004;13(2):93–121. 15297906 10.1002/mpr.168 PMC6878592

[34] Lawlor DA, Adamson J, Ebrahim S. Performance of the WHO Rose angina questionnaire in post-menopausal women: Are all of the questions necessary? Epidemiol Community Health. 2003;538–541. 10.1136/jech.57.7.538 PMC1732510 12821705

[35] Rose GA. The diagnosis of ischaemic heart pain and intermittent claudication in field surveys. Bull World Health Organ. 1962;27:645–58. 13974778 PMC2555832

[36] Oei H-HS, Vliegenthart R, Deckers JW, Hofman A, Oudkerk M, Witteman JCM. The association of Rose questionnaire angina pectoris and coronary calcification in a general population: The Rotterdam Coronary Calcification Study. Annals of Epidemiology. 2004;14(6):431–6. 10.1016/j.annepidem.2003.09.009 15246332

[37] R Core Team. R: A Language and Environment for Statistical Computing. 2014.

[38] Neale MC, Hunter MD, Pritikin JN, Zahery M, Brick TR, Kirkpatrick RM, et al OpenMx 2.0: Extended Structural Equation and Statistical Modeling. Psychometrika. 2015. 10.1007/s11336-014-9435-8 PMC4516707 25622929

[39] Rijsdijk FV, Sham PC. Analytic approaches to twin data using structural equation models. Briefings in bioinformatics. 2002;3(2):119–33. 12139432 10.1093/bib/3.2.119

[40] Dong Y, Peng CY. Principled missing data methods for researchers. SpringerPlus. 2013;2(1):222 10.1186/2193-1801-2-222 23853744 PMC3701793

[41] Burri A, Ogata S, Livshits G, Williams F. The Association between Chronic Widespread Musculoskeletal Pain, Depression and Fatigue Is Genetically Mediated. PloS one. 2015;10(11):e0140289 10.1371/journal.pone.0140289 26599910 PMC4657992

[42] Walsh SK, Hector EE, Andreasson AC, Jonsson-Rylander AC, Wainwright CL. GPR55 deletion in mice leads to age-related ventricular dysfunction and impaired adrenoceptor-mediated inotropic responses. PloS one. 2014;9(9):e108999 10.1371/journal.pone.0108999 25275556 PMC4183508

[43] Hocking LJ, Smith BH, Jones GT, Reid DM, Strachan DP, Macfarlane GJ. Genetic variation in the beta2-adrenergic receptor but not catecholamine-O-methyltransferase predisposes to chronic pain: results from the 1958 British Birth Cohort Study. Pain. 2010;149(1):143–51. 10.1016/j.pain.2010.01.023 20167428

[44] Smith SB, Maixner DW, Fillingim RB, Slade G, Gracely RH, Ambrose K, et al Large candidate gene association study reveals genetic risk factors and therapeutic targets for fibromyalgia. Arthritis and rheumatism. 2012;64(2):584–93. 10.1002/art.33338 21905019 PMC3237946

[45] Marco EM, Echeverry-Alzate V, Lopez-Moreno JA, Gine E, Penasco S, Viveros MP. Consequences of early life stress on the expression of endocannabinoid-related genes in the rat brain. Behavioural pharmacology. 2014;25(5–6):547–56. 10.1097/FBP.0000000000000068 25083571

[46] Reich CG, Mihalik GR, Iskander AN, Seckler JC, Weiss MS. Adolescent chronic mild stress alters hippocampal CB1 receptor-mediated excitatory neurotransmission and plasticity. Neuroscience. 2013;253:444–54. 10.1016/j.neuroscience.2013.08.066 24035826 PMC3827785

[47] Maccari S, Krugers HJ, Morley-Fletcher S, Szyf M, Brunton PJ. The consequences of early-life adversity: neurobiological, behavioural and epigenetic adaptations. Journal of neuroendocrinology. 2014;26(10):707–23. 10.1111/jne.12175 25039443

[48] Colman I, Jones PB, Kuh D, Weeks M, Naicker K, Richards M, et al Early development, stress and depression across the life course: pathways to depression in a national British birth cohort. Psychological medicine. 2014;44(13):2845–54. 10.1017/S0033291714000385 25066933

[49] Jones GT, Power C, Macfarlane GJ. Adverse events in childhood and chronic widespread pain in adult life: Results from the 1958 British Birth Cohort Study. Pain. 2009;143(1–2):92–6. 10.1016/j.pain.2009.02.003 19304391

[50] Slavich GM, Irwin MR. From stress to inflammation and major depressive disorder: a social signal transduction theory of depression. Psychological bulletin. 2014;140(3):774–815. 10.1037/a0035302 24417575 PMC4006295

[51] Generaal E, Vogelzangs N, Macfarlane GJ, Geenen R, Smit JH, Dekker J, et al Basal inflammation and innate immune response in chronic multisite musculoskeletal pain. Pain. 2014;155(8):1605–12. 10.1016/j.pain.2014.05.007 24813297

[52] Hiles SA, Baker AL, de Malmanche T, McEvoy M, Boyle M, Attia J. The role of inflammatory markers in explaining the association between depression and cardiovascular hospitalisations. Journal of behavioral medicine. 2015. 10.1007/s10865-015-9637-2 25835436

[53] Tomasdottir MO, Sigurdsson JA, Petursson H, Kirkengen AL, Krokstad S, McEwen B, et al Self Reported Childhood Difficulties, Adult Multimorbidity and Allostatic Load. A Cross-Sectional Analysis of the Norwegian HUNT Study. PloS one. 2015;10(6):e0130591 10.1371/journal.pone.0130591 26086816 PMC4472345

[54] Generaal E, Vogelzangs N, Macfarlane GJ, Geenen R, Smit JH, de Geus EJ, et al Biological stress systems, adverse life events and the onset of chronic multisite musculoskeletal pain: a 6-year cohort study. Ann Rheum Dis. 2015. 10.1136/annrheumdis-2014-206741 25902791

[55] Livshits G, Macgregor AJ, Gieger C, Malkin I, Moayyeri A, Grallert H, et al An omics investigation into chronic widespread musculoskeletal pain reveals epiandrosterone sulfate as a potential biomarker. Pain. 2015;156(10):1845–51. 10.1097/j.pain.0000000000000200 25915148 PMC4770329

[56] de Heer EW, Gerrits MM, Beekman AT, Dekker J, van Marwijk HW, de Waal MW, et al The Association of Depression and Anxiety with Pain: A Study from NESDA. PloS one. 2014;9(10):e106907 10.1371/journal.pone.0106907 25330004 PMC4198088

[57] Gerrits MM, van Oppen P, Leone SS, van Marwijk HW, van der Horst HE, Penninx BW. Pain, not chronic disease, is associated with the recurrence of depressive and anxiety disorders. BMC psychiatry. 2014;14:187 10.1186/1471-244X-14-187 24965597 PMC4090396

[58] Goldstein BI, Carnethon MR, Matthews KA, McIntyre RS, Miller GE, Raghuveer G, et al Major Depressive Disorder and Bipolar Disorder Predispose Youth to Accelerated Atherosclerosis and Early Cardiovascular Disease: A Scientific Statement From the American Heart Association. Circulation. 2015;132(10):965–86. 10.1161/CIR.0000000000000229 26260736

[59] Morgan MAJ, Coates MJ, Dunbar JA, Reddy P, Schlicht K, Fuller J. The TrueBlue model of collaborative care using practice nurses as case managers for depression alongside diabetes or heart disease: a randomised trial. BMJ Open. 2013;3(1). 10.1136/bmjopen-2012-002171 PMC3563126 23355671

[60] Bromley C DS, Gray L, Hughes T, Leyland AH, McNeill G, Marcinkiewicz A,. The Scottish Health Survey 2013: volume 1—Main Report.

[61] Elliott AM, Smith BH, Penny KI, Chambers WA, Smith WC. The epidemiology of chronic pain in the community. Lancet. 1999;354:1248–52. 10520633 10.1016/s0140-6736(99)03057-3

[62] Kroenke K, Bair MJ, Damush TM, Wu J, Hoke S, Sutherland J, et al Optimized antidepressant therapy and pain self-management in primary care patients with depression and musculoskeletal pain: a randomized controlled trial. JAMA. 2009;301:2099–110. 10.1001/jama.2009.723 19470987 PMC2884224

