# Supplementary material for: Chronic pain, depression and cardiovascular disease linked through a shared genetic predisposition: Analysis of a family-based cohort and twin study
Source: PLoS One. 2017 Feb 22;12(2):e0170653. doi: 10.1371/journal.pone.0170653 (PMC5321424; doi:10.1371/journal.pone.0170653)
Supplement: S1 Table — (PDF) [file pone.0170653.s001.pdf]

S1 Table. The effect of depression and/or angina on the occurrence of chronic pain in GS: SFHS overall and stratified according to gender.

Exposure	Outcome	Group	Unadjusted		Adjusted	
			N	OR [95% CI]	N	OR [95% CI]
**Chronic pain in the presence of depression**						
Depression	Chronic pain	Overall†	13, 376	2.80 [a] [2.52 to 3.11]	11,679	2.64[a] [2.34 to 2.97]
		Females only‡	7,845	2.57 [a] [2.27 to 2.91]	6,832	2.63[a] [2.29 to 3.03]
		Males only‡	5, 531	2.71 [a] [2.21 to 3.32]	4,847	2.61[a] [2.07 to 3.28]
**Chronic pain in the presence of angina**						
Angina	Chronic pain	Overall†	14,564	5.44 [a] [4.83 to 6.13]	11,973	4.19[a] [3.64 to 4.82]
		Females only‡	8,720	6.13 [a] [5.26 to 7.16]	7,193	4.95[a] [4.13 to 5.93]
		Males only‡	5,844	4.93 [a] [4.06 to 5.99]	4,780	3.20[a] [2.54 to 4.04]
**Chronic pain in the presence of depression and angina**						
Depression and angina	Chronic pain	Overall†	9,728	13.28 [a] [10.02 to 17.59]	8,543	9.43[a] [6.85 to 12.98]
		Females only‡	5,644	13.20 [a] [9.34 to 18.66]	4,956	10.24[a] [6.93 to 15.13]
		Males only‡	4,084	11.77 [a] [7.16 to 19.37]	3,587	8.01[a] [4.54 to 14.12]

†valid data adjusted for age, gender, education, SIMD and smoking status; overall results are also shown in Table 3

‡valid data adjusted for age, education, SIMD and smoking status;

[a]= $p<0.001$
