# Supplementary material for: Chronic pain, depression and cardiovascular disease linked through a shared genetic predisposition: Analysis of a family-based cohort and twin study
Source: PLoS One. 2017 Feb 22;12(2):e0170653. doi: 10.1371/journal.pone.0170653 (PMC5321424; doi:10.1371/journal.pone.0170653)
Supplement: S2 Table — (PDF) [file pone.0170653.s002.pdf]

S2 Table. The effect of comorbidity on the occurrence of depression and/or angina in GS: SFHS overall and stratified according to gender.

**Exposure**	**Outcome**	**Group**	**Unadjusted**		**Adjusted**	
			**N**	**OR [95% CI]**	**N**	**OR [95% CI]**
**Depression in the presence of chronic pain**						
Chronic Pain	Depression	Overall[†]	13,376	2.80[a] [2.52 to 3.11]	11,679	2.62[a] [2.32 to 2.96]
		Females only[‡]	7,845	2.57[a] [2.27 to 2.91]	6,832	2.63[a] [2.28 to 3.03]
		Males only[‡]	5,531	2.71[a] [2.21 to 3.32]	4,847	2.58[a] [2.04 to 3.25]
**Depression in the presence of angina**						
Angina	Depression	Overall[†]	16,224	2.18[a] [1.92 to 2.47]	14,121	2.10[a] [1.82 to 2.43]
		Females only[‡]	9,669	2.13[a] [1.82 to 2.48]	8,379	2.05[a] [1.72 to 2.45]
		Males only[‡]	6,555	2.43[a] [1.94 to 3.03]	5,742	2.20[a, d] [1.71 to 2.84]
**Depression in the presence of chronic pain and angina**						
Chronic pain and angina	Depression	Overall[†]	9,461	4.36[a] [3.58 to 5.30]	8,297	3.78[a] [2.99 to 4.78]
		Females only[‡]	5,480	3.91[a] [3.10 to 4.91]	4,805	3.76[a, b] [2.87 to 4.94]
		Males only[‡]	3,981	4.77[a] [3.26 to 6.97]	3,492	3.86[a, c] [2.49 to 5.99]
**Angina in the presence of chronic pain**						
Chronic pain	Angina	Overall[†]	14,564	5.45[a] [4.84 to 6.14]	11,973	4.23[a] [3.67 to 4.86]
		Females only[‡]	8,720	6.13[a] [5.26 to 7.16]	7,193	4.97[a] [4.16 to 5.95]
		Males only[‡]	5,844	4.93[a] [4.06 to 5.99]	4,780	3.31[a, e] [2.63 to 4.17]
**Angina in the presence of depression**						
Depression	Angina	Overall[†]	16,224	2.18[a] [1.92 to 2.47]	14,121	2.20[a] [1.90 to 2.54]
		Females only[‡]	9,669	2.13[a] [1.82 to 2.48]	8,379	2.11[a] [1.77 to 2.52]
		Males only[‡]	6,555	2.43[a] [1.94 to 3.03]	5,742	2.37[a, f] [1.83 to 3.06]
**Angina in the presence of chronic pain and depression**						
Chronic pain and depression	Angina	Overall[†]	9,003	9.15[a] [7.43 to 11.26]	7,919	7.76[a] [6.05 to 9.95]
		Females only[‡]	5,120	10.19[a] [7.91 to 13.12]	4,506	7.77[a] [5.79 to 10.42]

		Males only[‡]	3,883	9.85 [a] [6.52 to 14.88]	3,413	8.45 [a, g] [5.25 to 13.59]

[†]valid data adjusted for age, gender, education, SIMD and smoking status; overall results are also reported in Table 3; [‡]valid data adjusted for age, education, SIMD and smoking status;

a= p<0.001; b = Variable removed from model, SIMD P=0.62; c= Variable removed from model, Age P=0.21; d = Variable removed from model: education P=0.18; e = Variable removed from model: education P=0.80; f= Variable removed from model: education P=0.12; g = Variables removed from model: SIMD P=0.45 and Education P=0.30
