# Supplementary material for: Chronic pain, depression and cardiovascular disease linked through a shared genetic predisposition: Analysis of a family-based cohort and twin study
Source: PLoS One. 2017 Feb 22;12(2):e0170653. doi: 10.1371/journal.pone.0170653 (PMC5321424; doi:10.1371/journal.pone.0170653)
Supplement: S3 Table — (PDF) [file pone.0170653.s003.pdf]

S3 Table. The effect of chronic pain on the occurrence of depression and/or angina in the "unrelated" subgroup (n=9,163) and stratified according to gender.

Exposure	Outcome	Group	Unadjusted		Adjusted	
			N	OR [95% CI]	N	OR [95% CI]
**Chronic pain in the presence of depression**						
Depression	Chronic pain	Overall[†]	4,780	2.70 [a] [2.26 to 3.23]	4,179	2.46[a] [2.02 to 3.01]
		Females only[‡]	2,743	2.64 [a] [2.13 to 3.26]	2,385	2.66 [a] [2.09 to 3.37]
		Males only[‡]	2,037	2.25 [a] [1.59 to 3.17]	1,794	2.10 [a, b] [1.44 to 3.07]
**Chronic pain in the presence of angina**						
Angina	Chronic pain	Overall[†]	5,113	4.26 [a] [3.60 to 5.03]	4,267	3.70 [a] [3.04 to 4.51]
		Females only[‡]	3,006	5.04 [a] [4.03 to 6.30]	2,505	4.54 [a] [3.50 to 5.89]
		Males only[‡]	2,107	3.64 [a] [2.79 to 4.75]	1,762	2.87 [a, c] [2.11 to 3.91]
**Chronic pain in the presence of depression and angina**						
Depression and angina	Chronic pain	Overall[†]	3,380	12.31 [a] [7.83 to 19.35]	2,967	8.70 [a] [5.23 to 14.46]
		Females only[‡]	1,922	13.76 [a] [7.73 to 24.48]	1,678	12.78 [a] [6.47 to 25.25]
		Males only[‡]	1,458	8.71 [a] [4.06 to 18.69]	1,289	5.20 [a, d] [2.25 to 12.04]

[†]valid data adjusted for age, gender, education, SIMD and smoking status; [‡]valid data adjusted for age, education, SIMD and smoking status; a= p<0.001; b= Variables not in final model: age (P=0.97) and smoking status (P=0.17) ; c= Variables not in final model: age (P= 0.86); smoking status (P=0.13); d= Variables not in final model: age (P= 0.89); smoking status (P=0.50)
