# Supplementary material for: Chronic pain, depression and cardiovascular disease linked through a shared genetic predisposition: Analysis of a family-based cohort and twin study
Source: PLoS One. 2017 Feb 22;12(2):e0170653. doi: 10.1371/journal.pone.0170653 (PMC5321424; doi:10.1371/journal.pone.0170653)
Supplement: S4 Table — (PDF) [file pone.0170653.s004.pdf]

S4 Table. The effect of depression on the occurrence of chronic pain and/or angina in the "unrelated" subgroup (n=9,163) and stratified according to gender.

Exposure	Outcome	Group	Unadjusted		Adjusted	
			N	OR [95% CI]	N	OR [95% CI]
**Depression in the presence of chronic pain**						
Chronic Pain	Depression	Overall†	4,780	2.70 [a] [2.26 to 3.23]	4,179	2.46 [a, b] [2.02 to 3.01]
		Females only‡	2,743	2.64 [a] [2.13 to 3.26]	2,385	2.74 [a ,c] [2.16 to 3.46]
		Males only‡	2,037	2.25 [a] [1.59 to 3.17]	1,794	2.05 [a, d] [1.41 to 2.98]
**Depression in the presence of angina**						
Angina	Depression	Overall†	6,264	2.08 [a] [1.73 to 2.51]	5,453	2.08 [a] [1.67 to 2.60]
		Females only‡	3,662	2.08 [a] [1.66 to 2.62]	3,165	2.07 [a, e] [1.59 to 2.69]
		Males only‡	2,602	2.28 [a] [1.63 to 3.21]	2,288	2.25 [a, f] [1.52 to 3.33]
**Depression in the presence of chronic pain and angina**						
Chronic pain and angina	Depression	Overall†	3,149	4.33 [a] [3.23 to 5.81]	2,769	4.23 [a, g] [3.00 to 5.96]
		Females only‡	1,783	4.10 [a] [2.90 to 5.80]	1,566	4.77 [a, h] [3.23 to 7.05]
		Males only‡	1,366	4.12 [a] [2.34 to 7.27]	1,203	3.96 [a, i] [2.05 to 7.66]

†valid data adjusted for age, gender, education, SIMD and smoking status; ‡valid data adjusted for age, education, SIMD and smoking status; a= p<0.001; b= Variables not in final model: Education (P=0.14); c= Variables not in final model: Education (P=0.38); SIMD (P=0.21); d= Variables not in final model: Education (P=0.20); SIMD (P=0.48); e= Variables not in final model: SIMD (P=0.30); f= Variables not in final model: Education (P=0.40); g= Variables not in final model: SIMD (P= 0.12); Education (P=0.28); h= Variables not in final model: Smoking (P=0.10); SIMD (P=0.34); Education (P=0.22); i= Variables not in final model: SIMD (P=0.17); Education (P=0.94)
