# Supplementary material for: Chronic pain, depression and cardiovascular disease linked through a shared genetic predisposition: Analysis of a family-based cohort and twin study
Source: PLoS One. 2017 Feb 22;12(2):e0170653. doi: 10.1371/journal.pone.0170653 (PMC5321424; doi:10.1371/journal.pone.0170653)
Supplement: S5 Table — (PDF) [file pone.0170653.s005.pdf]

S5 Table. The effect of angina on the occurrence of depression and/or chronic pain in the "unrelated" subgroup (n=9,163) and stratified according to gender.

Exposure	Outcome	Group	Unadjusted		Adjusted	
			N	OR [95% CI]	N	OR [95% CI]
**Angina in the presence of chronic pain**						
Chronic pain	Angina	Overall†	5,113	4.26 [a] [3.60 to 5.03]	4,267	3.76 [a] [3.09 to 4.58]
		Females only‡	3,006	5.04 [a] [4.03 to 6.30]	2,505	4.61 [a] [3.56 to 5.98]
		Males only‡	2,107	3.64 [a] [2.79 to 4.75]	1,762	3.06 [a, b] [2.23 to 4.19]
**Angina in the presence of depression**						
Depression	Angina	Overall†	6,264	2.08 [a] [1.73 to 2.51]	5,453	2.25[a] [1.80 to 2.80]
		Females only‡	3,662	2.08 [a] [1.66 to 2.62]	3,165	2.09[a] [1.60 to 2.73]
		Males only‡	2,602	2.28 [a] [1.63 to 3.21]	2,288	2.52 [a, c] [1.71 to 3.72]
**Angina in the presence of chronic pain and depression**						
Chronic pain and depression	Angina	Overall†	3,054	7.32 [a] [5.35 to 10.00]	2,695	8.13 [a, d] [5.58 to 11.84]
		Females only‡	1,698	8.13 [a] [5.58 to 11.86]	1,493	7.66 [a, e] [4.97 to 11.80]
		Males only‡	1,356	9.05 [a] [4.68 to 17.47]	1,202	10.33 [a, f] [4.89 to 21.82]

†valid data adjusted for age, gender, education, SIMD and smoking status; ‡valid data adjusted for age, education, SIMD and smoking status; a= p<0.001; b= Variables not in final model: Smoking status (P=0.15); Education (P=0.47); c= Variables not in final model: Smoking status (P=0.26); d= Variables not in final model: Smoking status (P=0.12); Education (P=0.12); e= Variables not in final model: Smoking status (P=0.17); Education (P=0.14); f= Variables not in final model: Smoking status (P=0.28); Education (P=0.31); SIMD (P=0.18)
