# Supplementary material for: Chronic pain, depression and cardiovascular disease linked through a shared genetic predisposition: Analysis of a family-based cohort and twin study
Source: PLoS One. 2017 Feb 22;12(2):e0170653. doi: 10.1371/journal.pone.0170653 (PMC5321424; doi:10.1371/journal.pone.0170653)
Supplement: S6 Table — (PDF) [file pone.0170653.s006.pdf]

S6 Table. Unadjusted and adjusted ORs for co-occurrence of the same trait within sibling-pairs in GS:SFHS

Exposure (**Sib1** status)	Outcome (**Sib2** status)	Unadjusted		Adjusted		λ_S
		N	OR [95% CI]	N	OR [95% CI]	
Angina	Angina	4,866	3·21 [2·42 – 4·26] ***	3,989	2·78 [2·00 – 3·86] ***	2·31 [1·86 – 2·88]*
Depression	Depression	4,425	2·25 [1·84 – 2·75] ***	3,803	2·16 [1·73 – 2·70] ***	1·65 [1·44 – 1·90]*
Chronic pain	Chronic pain	3,415	3·30 [2·73 – 4·00] ***	2,773	2·30 [1·83 – 2·89]***	1·84 [1·65 – 2·06]*

λ_S = sibling recurrence risk ratio; * p ≤0.05, ** p ≤0.01 & *** p ≤0.001
