# Supplementary material for: Chronic pain, depression and cardiovascular disease linked through a shared genetic predisposition: Analysis of a family-based cohort and twin study
Source: PLoS One. 2017 Feb 22;12(2):e0170653. doi: 10.1371/journal.pone.0170653 (PMC5321424; doi:10.1371/journal.pone.0170653)
Supplement: S7 Table — (PDF) [file pone.0170653.s007.pdf]

S7 Table. Unadjusted and adjusted ORs of angina, depression and chronic pain within in sib1 subset.

Exposure (Sib1 status)	Outcome (Sib1 status)	Gender	Unadjusted		Adjusted	
			N	OR	N	OR
Angina	Chronic pain	Overall	4,042	5.87 [4.56 to 7.55] ***	3,304	4.71 [3.52 to 6.31] ***
		Female-only sib pairs	2,451	6.38 [4.55 to 8.95] ***	2,001	4.98 [3.39 to 7.31] ***
		Male-only sib pairs	1,584	6.24 [4.19 to 9.28] ***	1,303	4.28 [2.70 to 6.78] ***
Angina	Depression	Overall	As "depression-angina"		3,875	1.69 [1.25 to 2.29] ***
		Female-only sib pairs			2,320	1.43 [0.9 to 2.12]
		Male-only sib pairs			1,555	2.23 [1.38 to 3.62] ***
Chronic pain	Angina	Overall	As "angina-chronic pain"		3,304	4.69 [3.50 to 6.28] ***
		Female-only sib pairs			2,001	4.98 [3.40 to 7.30] ***
		Male-only sib pairs			1,303	4.35 [2.76 to 6.87] ***
Chronic pain	Depression	Overall	3,716	2.89 [2.36 to 3.54] ***	3,225	2.48 [1.96 to 3.13] ***
		Female-only sib pairs	2,214	2.53 [1.99 to 3.22] ***	1,915	2.33 [1.76 to 3.07] ***
		Male-only sib pairs	1,494	3.37 [2.32 to 4.90] ***	1,310	2.94 [1.90 to 4.54] ***
Depression	Angina	Overall	4,470	1.95 [1.50 to 2.54] ***	3,875	1.71 [1.26 to 2.32] ***
		Female-only sib pairs	2,689	1.67 [1.19 to 2.35] **	2,320	1.42 [0.96 to 2.11]
		Male-only sib pairs	1,771	2.78 [1.83 to 4.24] ***	1,555	2.42 [1.49 to 3.92] ***
Depression	Chronic pain	Overall	As "chronic pain-depression"		3,225	2.52 [2.00 to 3.18] ***
		Female-only sib pairs			1,915	2.35 [1.78 to 3.10] ***
		Male-only sib pairs			1,310	3.04 [1.9 to -4.68] ***

** p≤0.01, *** p ≤0.001
