# Supplementary material for: Chronic pain, depression and cardiovascular disease linked through a shared genetic predisposition: Analysis of a family-based cohort and twin study
Source: PLoS One. 2017 Feb 22;12(2):e0170653. doi: 10.1371/journal.pone.0170653 (PMC5321424; doi:10.1371/journal.pone.0170653)
Supplement: S8 Table — (PDF) [file pone.0170653.s008.pdf]

S8 Table. Unadjusted and adjusted ORs for co-occurrence of the same trait overall and within same-gender sibling-pairs.

Exposure (Sib1 status)	Outcome (Sib2 status)	Gender	Unadjusted		Adjusted		λ_S
			N	OR [95% CI]	N	OR [95% CI]	
Angina	Angina	Overall†	4,866	3.21 [2.42 to 4.26] ***	3,989	2.78 [2.00 to 3.86] ***	2.31 [1.86 to 2.88]*
		Female-only sib pairs	1,808	3.89 [2.44 to 6.21] ***	1,485	3.31 [1.94 to 5.63] ***	
		Male-only sib pairs	788	3.37 [1.68 to 6.76] ***	648	2.91 [1.29 to 6.57] **	
Depression	Depression	Overall†	4,425	2.25 [1.84 to 2.75] ***	3,803	2.16 [1.73 to 2.70] ***	1.65 [1.44 to 1.90]*
		Female-only sib pairs	1,641	2.83 [2.13 to 3.77] ***	1,393	2.97 [2.16 to 4.08] ***	
		Male-only sib pairs	766	2.21 [1.15 to 4.24] *	666	2.19 [1.10 to 4.37] *	
Chronic pain	Chronic pain	Overall†	3,415	3.30 [2.73 to 4.00] ***	2,773	2.30 [1.83 to 2.89] ***	1.84 [1.65 to 2.06]*
		Female-only sib pairs	1,276	3.54 [2.65 to 4.73] ***	1,037	2.66 [1.89 to 3.75] ***	
		Male-only sib pairs	575	3.15 [1.75 to 5.67] ***	464	2.21 [1.09 to 4.48] *	

λ_S = sibling recurrence risk ratio; * p ≤0.05;** p ≤0.01; *** p ≤0.001; †overall results are also shown in Table 4
