# Supplementary material for: Chronic pain, depression and cardiovascular disease linked through a shared genetic predisposition: Analysis of a family-based cohort and twin study
Source: PLoS One. 2017 Feb 22;12(2):e0170653. doi: 10.1371/journal.pone.0170653 (PMC5321424; doi:10.1371/journal.pone.0170653)
Supplement: S9 Table — (PDF) [file pone.0170653.s009.pdf]

S9 Table. Unadjusted and adjusted ORs for cross-trait analysis of angina, depression and chronic pain overall and within same-gender sibling-pairs.

Exposure (Sib1 status)	Outcome (Sib2 status)	Gender	Unadjusted		Adjusted	
			N	OR	N	OR
Angina	Chronic pain	Overall†	4,014	2.61 [2.03 to 3.37] ***	3,275	2.19 [1.63 to 2.95] ***
		Female-only sib pairs	1,475	2.36 [1.55 to 3.60] ***	1,207	2.12 [1.32 to 3.38] **
		Male-only sib pairs	665	2.02 [1.03 to 3.92] *	537	0.96 [0.40 to 2.29]
Angina	Depression	Overall†	4,595	1.52 [1.16 to 1.99] **	3,967	1.48 [1.09 to 2.01] **
		Female-only sib pairs	1,704	1.23 [0.80 to 1.90]	1,450	1.08 [0.65 to 1.78]
		Male-only sib pairs	768	1.44 [0.66 to 3.16]	672	1.43 [0.63 to 3.28]
Chronic pain	Angina	Overall†	4,026	2.48 [1.93 to 3.19] ***	3,280	2.01 [1.49 to 2.71] ***
		Female-only sib pairs	1,514	2.49 [1.67 to 3.73] ***	1,234	2.38 [1.47 to 3.84] ***
		Male-only sib pairs	660	2.41 [1.17 to 4.98] *	542	2.26 [0.96 to 5.31]
Chronic pain	Depression	Overall†	3,831	1.84 [1.50 to 2.26] ***	3,295	1.69 [1.34 to 2.14] ***
		Female-only sib pairs	1,428	1.69 [1.25 to 2.27] ***	1,210	1.53 [1.08 to 2.16] *
		Male-only sib pairs	643	2.24 [1.19 to 4.21] **	562	2.09 [1.04 to 4.17] *
Depression	Angina	Overall†	4,562	1.97 [1.54 to 2.54] ***	3,757	1.98 [1.49 to 2.65] ***
		Female-only sib pairs	1,704	1.97 [1.32 to 2.94] ***	1,406	2.03 [1.29 to 3.20] **
		Male-only sib pairs	769	2.92 [1.53 to 5.57] ***	634	2.38 [1.18 to 5.06] *
Depression	Chronic pain	Overall†	3,796	1.30 [1.05 to 1.61] *	3,124	1.34

						[1.05 to 1.71] *
		Female-only sib pairs	1,405	1.42 [1.04 to 1.93] *	1,159	1.40 [0.98 to 1.99]
		Male-only sib pairs	659	1.60 [0.86 to 2.96]	537	1.61 [0.79 to 3.25]

λ_S = sibling recurrence risk ratio ; p≤0.05; ** p≤0.01;*** p ≤0.001; †overall results are also shown in Table 5.
