# Supplementary material for: Chronic pain, depression and cardiovascular disease linked through a shared genetic predisposition: Analysis of a family-based cohort and twin study
Source: PLoS One. 2017 Feb 22;12(2):e0170653. doi: 10.1371/journal.pone.0170653 (PMC5321424; doi:10.1371/journal.pone.0170653)
Supplement: S10 Table — (PDF) [file pone.0170653.s010.pdf]

S10 Table. Model comparison between the full ACE Cholesky and the best balance of model fit with parsimony for chronic pain[±] and cardiovascular disease (n=2,902): Twins UK

	Difference of log likelihood	Difference of degree of freedom	P-value	AIC	BIC
Full ACE Cholesky model	Base	Base	NA	-5919.404	-36506.49
AE Cholesky model	5.226656	3	0.16	-5920.177	-36523.1
Best fitting model (Fig 1)	6.476206	4	0.17	-5920.928	-36529.14

A, additive genetic factors; C, shared environmental factors; E, non-shared environmental factors; D, non-additive genetic factors; [±]chronic widespread pain; AIC, Akaike information criterion; BIC, Schwarz's Bayesian information criteria; NA, not applicable.
